# P-2356. A SFGM-TC real-world study to characterize pediatric patients undergoing allogeneic Hematopoietic Stem Cell Transplantation (HSCT) and identify the cytomegalovirus (CMV) serology status of both recipients and their donors

**DOI:** 10.1093/ofid/ofae631.2507

**Published:** 2025-01-29

**Authors:** Xavier Bourge, Guilhem Tournaire, Jean-Hugues Dalle, Bénédicte Neven, Marie Ouachée-Chardin, Arthur Sterin, Charlotte Jubert, Bénédicte Bruno, Virginie Gandemer, Cécile Pochon, Fanny Rialland, Catherine Paillard, Marie Robin, Anne Sirvent, Nimrod Buchbinder, Dominique Plantaz, Justyna Kanold, Gaelle Guillerm, Etienne Daguindau, Anne Huynh, Amandine Charbonnier, Felipe Suarez, Natacha Maillard, Sylvie François, Jean-Valère Malfuson, Raynier Devillier, Nicole Raus, Stephanie Nguyen

**Affiliations:** MSD France, VOURLES, Rhone-Alpes, France; MSD France, VOURLES, Rhone-Alpes, France; AP-HP, Paris, Ile-de-France, France; AP-HP, Paris, Ile-de-France, France; IHOP, LYON, Rhone-Alpes, France; AP-HM, Marseille, Provence-Alpes-Cote d'Azur, France; CHU Bordeaux, Bordeaux, Aquitaine, France; CHU Lille, Lille, Nord-Pas-de-Calais, France; CHU Rennes, Rennes, Bretagne, France; CHRU Nancy, Nancy, Lorraine, France; CHU Nantes, Nantes, Pays de la Loire, France; CHU Strasbourg, Strasbourg, Alsace, France; AP-HP, Paris, Ile-de-France, France; CHU Montpellier, Montpellier, Languedoc-Roussillon, France; CHU Rouen, Rouen, Haute-Normandie, France; CHU Grenoble, Grenoble, Rhone-Alpes, France; CHU Clermont-Ferrand, Clermont-Ferrand, Auvergne, France; CHU Brest, Brest, Bretagne, France; CHU Besancon, Besancon, Franche-Comte, France; Toulouse Oncopole, Toulouse, Languedoc-Roussillon, France; CHU Amiens, Amiens, Nord-Pas-de-Calais, France; AP-HP, Paris, Ile-de-France, France; CHU Poitiers, Poitier, Pays de la Loire, France; CHU Angers, Angers, Pays de la Loire, France; AP-HP, Paris, Ile-de-France, France; IPC, Marseille, Provence-Alpes-Cote d'Azur, France; CHU Lyon, LYON, Rhone-Alpes, France; APHP, Paris, Ile-de-France, France

## Abstract

**Background:**

The aim of this study was to perform a description of pediatric patients’ characteristics undergoing an allogeneic Hematopoietic Stem Cell Transplantation (HSCT) and to identify their cytomegalovirus (CMV) serology status and that of their donors

**Figure:**
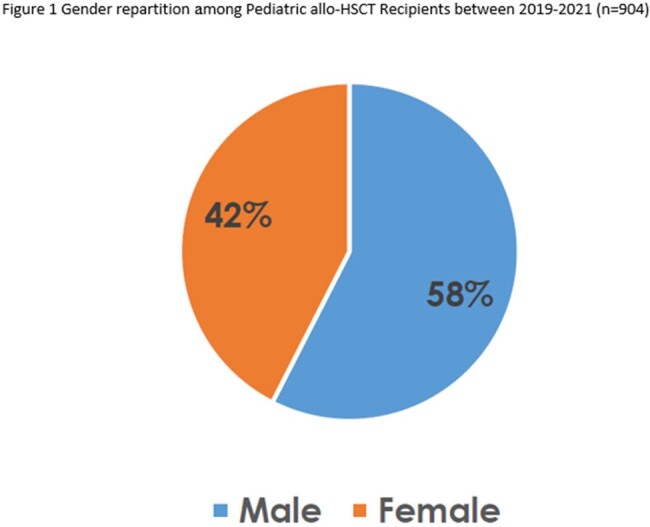

**Methods:**

Patients under the age of 18 who received an allogeneic HSCT in a French center reporting data in the ProMISe database of the francophone society of bone marrow transplant and cellular therapy (SFGM-TC) during the period comprised between January 1, 2019, and December 31, 2021

**Figure:**
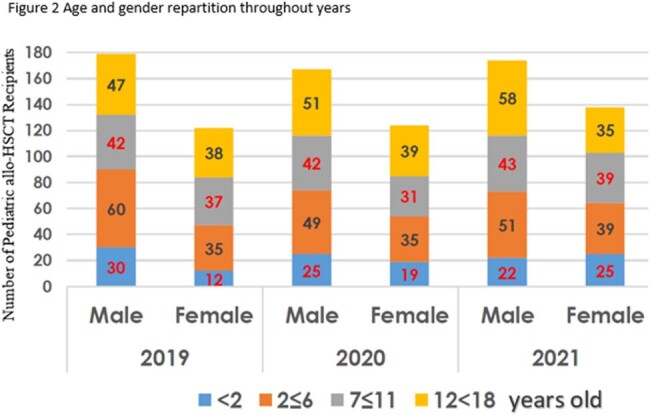

**Results:**

904 allo-HSCT were performed between January 1, 2019, and December 31, 2021, in 22 hematopoietic stem cell transplant centres in France. Among them 57,5% (520) were males (Figure 1). Patients ages were analysed with 14,7% (133) being strictly below 2 years old, 29,8% (269) being between 2 and 6 years old, 25,9% (234) being between 7 and 11 years old and 29,7% (268) being between 12 and 18 years old. Age classes distribution wasn’t different throughout the years analysed, as shown in Figure 2

CMV serostatus of both donor and recipient were available for 96% of patients. Donor negative and Recipient negative (D-/R-) accounted for 32% of the population, Donor positive and Recipient negative (D+/R-) accounted for 16%, Donor Positive and Recipient positive (D+/R+) accounted for 29% and lastly Donor negative and Recipient positive (D-/R+) accounted for 19%. Serostatus was analysed through age classes (Table 1) showing a numerical trend toward a higher CMV seropositivity for recipient being older than 2 years old. R+ patients were 42,1% below 2 years old and 49,3% in the 2 to 6-year-old age group. The percentage remained stable in older patients.

The primary diagnoses leading to the graft were mainly primary immune deficiency for patients under 2 years old, accounting for 45.1% (60/133). After 2 years old, the leading cause was acute leukaemia (Table 2). Pediatric patients mainly received bone marrow (71%) as the source of stem cells, followed by cord blood (18%) and peripheral blood (11%).

**Table 1:**
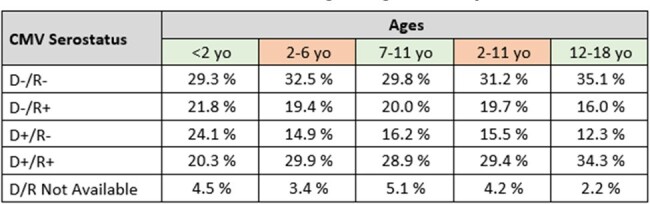
CMV Serostatus according to age of recipients

**Conclusion:**

The diagnosis leading to HSCT in the pediatric population differs according to age class. Serostatus also differs according to age class, with older patients approaching the levels of seropositivity seen in adults.

**Figure:**
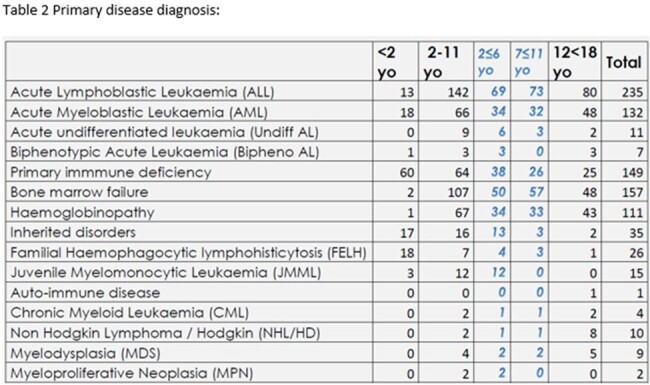

**Disclosures:**

Xavier Bourge, PharmD, MSD France: Employee Guilhem Tournaire, MD, MSD France: Employee Jean-Hugues Dalle, MD, MSD France: Advisor/Consultant Bénédicte Neven, MD, MSD France: Advisor/Consultant Marie Robin, MD, MSD France: Grant/Research Support Raynier DEVILLIER, MD, MSD France: Grant/Research Support Nicole Raus, n/a, MSD France: Grant/Research Support Stephanie Nguyen, n/a, MSD France: Grant/Research Support

